# Effects of body size and countermeasure exercise on estimates of life support resources during all-female crewed exploration missions

**DOI:** 10.1038/s41598-023-31713-6

**Published:** 2023-04-12

**Authors:** Jonathan P. R. Scott, David A. Green, Guillaume Weerts, Samuel N. Cheuvront

**Affiliations:** 1 Institut Médecine Physiologie Spatiale (MEDES), Toulouse, France; 2 Space Medicine Team, European Astronaut Centre, European Space Agency, Cologne, Germany; 3 KBR GmbH, Cologne, Germany; 4 Centre of Human and Applied Physiological Sciences, King’s College London, London, UK; 5 Sports Science Synergy, LLC, Franklin, MA USA

**Keywords:** Metabolism, Homeostasis, Physiology

## Abstract

Employing a methodology reported in a recent theoretical study on male astronauts, this study estimated the effects of body size and aerobic countermeasure (CM) exercise in a four-person, all-female crew composed of individuals drawn from a stature range (1.50- to 1.90-m) representative of current space agency requirements (which exist for stature, but not for body mass) upon total energy expenditure (TEE), oxygen (O_2_) consumption, carbon dioxide (CO_2_) and metabolic heat (H_prod_) production, and water requirements for hydration, during space exploration missions. Assuming geometric similarity across the stature range, estimates were derived using available female astronaut data (mean age: 40-years; BMI: 22.7-kg·m^−2^; resting VO_2_ and VO_2max_: 3.3- and 40.5-mL·kg^−1^·min^−1^) on 30- and 1080-day missions, without and with, ISS-like countermeasure exercise (modelled as 2 × 30-min aerobic exercise at 75% VO_2max_, 6-day·week^−1^). Where spaceflight-specific data/equations were not available, terrestrial equivalents were used. Body size alone increased 24-h TEE (+ 30%), O_2_ consumption (+ 60%), CO_2_ (+ 60%) and H_prod_ (+ 60%) production, and water requirements (+ 17%). With CM exercise, the increases were + 25–31%, + 29%, + 32%, + 38% and + 17–25% across the stature range. Compared to the previous study of theoretical male astronauts, the effect of body size on TEE was markedly less in females, and, at equivalent statures, all parameter estimates were lower for females, with relative differences ranging from -5% to -29%. When compared at the 50th percentile for stature for US females and males, these differences increased to − 11% to − 41% and translated to larger reductions in TEE, O_2_ and water requirements, and less CO_2_ and H_prod_ during 1080-day missions using CM exercise. Differences between female and male theoretical astronauts result from lower resting and exercising O_2_ requirements (based on available astronaut data) of female astronauts, who are lighter than male astronauts at equivalent statures and have lower relative VO_2max_ values. These data, combined with the current move towards smaller diameter space habitat modules, point to a number of potential advantages of all-female crews during future human space exploration missions.

## Introduction

Human basal metabolism is, in absolute terms, proportional with body size, with larger individuals possessing higher resting oxygen consumption (VO_2_), carbon dioxide production (VCO_2_) and metabolic heat production^1^. These differences persist when metabolism is elevated during physical activity (e.g., 75% maximal oxygen uptake [VO_2max_]), assuming equal aerobic fitness (VO_2max_ relative to body mass)^2^. In most occupational settings, these differences do not have significant implications. Even in closed terrestrial (i.e., artificially sustained) environments, such differences have minimal impact, as long exposure times occur in relatively large and well-resourced environments (e.g., submarines), or in highly confined environments exposures are short and crew activity levels are relatively low (e.g., operating submersibles).

Current missions to the International Space Station (ISS) in Low Earth Orbit (LEO) combine an artificially sustained closed environment with relatively high levels of physical activity due to daily countermeasure (CM) exercise^3^, performed in an attempt to ameliorate multi-system adaptation associated with prolonged spaceflight^4^. Regular (7–8 per year) re-supply of food and life support resources to LEO is currently provided for NASA by SpaceX and Northrup Grumman, in addition to Rocosmos’s ‘Progress’ and JAXA’s ‘HTV’ spacecraft. Beyond LEO, however, such as Artemis missions to Gateway and the Lunar surface^5^, re-supply will be significantly more difficult, with even greater challenges as exploration missions go beyond the Moon to destinations such as Mars.

Total energy intake by ISS astronauts is reportedly as much as ~ 20–25% below recommended levels^6,7^, with overall in-flight physical activity associated with spaceflight-induced total energy expenditure (TEE) and body composition changes and thus energy requirements^6^. As a result, during future exploration missions, where CM exercise programmes with high levels of physical activity (and thus energy expenditure) will be combined with more restrictive life-support constraints^8^, crewmember total metabolic activity may become a critical mission planning consideration^9^. Given this, we^9^ estimated the implications of body size (across a stature range of 1.50- to 1.90-m) and the use of CM exercise in an all-male crew upon mission resources. This study estimated that increasing stature from 1.50-m to 1.90-m increased (+ 44%) 24-h TEE, O_2_ consumption, and CO_2_ and metabolic heat production (+60%), and water required for hydration (+ 19%), with ISS-like CM exercise further increasing TEE (+ 29–32%), O_2_ consumption (+ 31%), CO_2_ (+ 35%) and metabolic heat (+ 42%) production, and water requirements (+ 23–33%) across the stature range.

The previous study did not evaluate the effect of body size and the use of CM exercise in females, yet, the first orbital spaceflight by a female (Valentina Tereshkova) in Vostok 6 on June 16th, 1963 was just two years after the first male Soviet cosmonaut, Yuri Gagarin (April 12th, 1961). In the United States (US), Brigadier General Donald Flickinger and Dr. W. Randolph Lovelace II, the NASA medical staff that tested male candidate pilots for the Mercury 7 space programme, considered the inclusion of women as early as 1959. As described in Ryan et al.^10^, Flickinger and Lovelace II identified a number of potential benefits of flying females. These included (compared to males) lower body mass (and thus reducing the fuel required to reach orbit), lower oxygen consumption^11^, as well as a lower risk of heart attack, a belief that the female reproductive organs were less susceptible to ionizing radiation, and the suggestion that females had better tolerance to cramped spaces and prolonged isolation^12,13^.

Although the ‘Woman in Space Earliest’ (WISE) Programme, established by Flickinger with the aim of extending the testing that had been undertaken by male candidates for the Mercury programme, was cancelled, Flickinger’s continued efforts resulted in the private ‘Woman in Space Programme’. The programme was not officially sanctioned and, therefore, not supported by government facilities, requiring the use of other laboratories^10^. Under this programme 19 women underwent physical examination and extensive physiological and psychological testing, identical to that required of official male candidates^14,15^, 13 of which passed with “no medical reservations”^16^. Data from the VO_2max_ tests showed that the relative VO_2max_ values of the top four females were comparable to the average from 267 similarly aged male pilots^17^. This population was from which NASA astronauts (all male) were exclusively drawn due to candidates being required to be jet pilots who had graduated from a military test pilot school and had at least 1500-h of flying time, at a time when women were barred from military test pilot schools. These four females were shorter in stature (168 ± 3-cm *vs*. 178 ± 1-cm), and had a lower body mass (54 ± 2-kg *vs*. 76 ± 1-kg) and absolute VO_2max_ (1.73 ± 0.05 L·min^−1^ *vs*. 2.57 ± 0.04 L·min^−1^) than the male group^10^. Such sex differences persist in International Space Station (ISS) astronauts^18^.

To date (as of March 2022), only 75 women have flown into space^19^, representing < 10% of the astronaut population (between 1961 and March 2020^20^), but this situation is changing, with 50% (9/18) of those selected by NASA in 2020 to prepare for the Artemis programme^21^ being female. Thus, female astronauts are set to play a significant role in future space exploration missions. Given that female astronauts are, on average, of shorter stature than their male counterparts^18^, and the differences between males and females in terms of stature and body mass, aerobic fitness^22^, body composition^23^, and resting^24^, exercise-related^25^ and post-prandial^26^ metabolism, as well as a spectrum of potential physiological and behavioural responses to the spaceflight environment and/or its analogues^27^, it is critical to consider whether the sex of the crew has an operationally meaningful effect upon estimated mission resources.

Thus, this study builds upon previous work considering males^9^ to estimate the effect of body size (indexed from stature as this is currently a key operational anthropometric criterion, unlike body mass) and CM exercise upon TEE, O_2_ consumption, CO_2_ and metabolic heat production, and water requirements, during space exploration missions in an all-female crew. This paper considered two hypothetical scenarios:

1. Female crew living in microgravity, but performing no in-flight CM exercise.
2. Female crew living in microgravity and performing CM exercise comparable in volume to that currently employed on ISS^3^.

To examine the effect of sex on the increase in resource requirements with body size and CM exercise, the estimates generated for theoretical female astronauts are qualitatively compared to estimates from the previous paper on theoretical male astronauts^9^.

## Results

### Characteristics of theoretical astronaut population

Based on the study assumptions and calculations (see Methods), the characteristics of the theoretical female astronaut populations were generated (Table 1).

**Table 1:** Characteristics of the theoretical female and male (in brackets) astronaut populations (see^9^).

Stature (m)	1.50	1.60	1.70	1.80	1.90
BM (kg)	51.1 (59.6)	58.1 (67.8)	65.6 (76.6)	73.5 (85.9)	81.9 (95.7)
BSA (m^2^)	1.45 (1.54)	1.60 (1.71)	1.76 (1.88)	1.93 (2.06)	2.10 (2.24)
VO_2max_ (L·min^−1^)	2.07 (2.59)	2.35 (2.94)	2.66 (3.32)	2.98 (3.73)	3.32 (4.15)
Rest					
RMR (MJ·day^−1^)	5.07 (5.78)	5.47 (6.44)	5.89 (7.13)	6.33 (7.85)	6.78 (8.60)
NEAT (MJ·day^−1^)	2.03 (2.31)	2.19 (2.58)	2.36 (2.85)	2.53 (3.14)	2.71 (3.44)
VO_2_ (L·min^−1^)	0.169 (0.197)	0.192 (0.224)	0.216 (0.253)	0.243 (0.283)	0.270 (0.316)
VCO_2_ (L·min^−1^)	0.133 (0.155)	0.151 (0.176)	0.171 (0.199)	0.191 (0.223)	0.213 (0.249)
Basal M_prod_ (J·s^−1^)	57.5 (65.7)	65.4 (74.8)	73.8 (84.4)	82.8 (94.7)	92.2 (105.5)
Basal fluid needs (L·d^−1^)	2.51 (2.63)	2.61 (2.74)	2.71 (2.86)	2.82 (2.99)	2.94 (3.13)
Exercise @ 75% VO_2max_					
VO_2_ (L·min^−1^)	1.55 (1.94)	1.77 (2.21)	1.99 (2.49)	2.23 (2.79)	2.49 (3.11)
VCO_2_ (L·min^−1^)	1.35 (1.74)	1.53 (1.98)	1.73 (2.24)	1.94 (2.51)	2.16 (2.80)
EE (kcal·min^−1^)	7.6 (9.6)	8.7 (10.9)	9.8 (12.3)	11.0 (13.8)	12.2 (15.4)
M_prod_ (J·s^−1^)	529 (667)	602 (759)	680 (857)	762 (960)	849 (1070)
SR (mL·min^−1^)	7.2 (10.1)	8.3 (11.7)	9.6 (13.4)	10.9 (15.2)	12.4 (17.1)

*BM* body mass, *BSA* body surface area, *VO*_*2max*_ maximal rate of oxygen uptake, *RMR* resting metabolic rate, *NEAT* non-exercise activity thermogenesis, *VO*_*2*_ rate of oxygen consumption, *VCO*_*2*_ rate of carbon dioxide production, *M*_*prod*_ rate of metabolic heat production, *EE* energy expenditure, *SR* sweat rate. See main text for definition of assumptions.

### Responses to an acute bout of aerobic CM exercise

Female body size increased EE, O_2_ consumption, in addition to CO_2_ and heat production, by 60% across the stature range during a *single* bout (30-min at 75% VO_2max_) of aerobic CM exercise, whilst water requirements increased by 72% (Table 2).

**Table 2:** Estimated total energy expenditure (EE), oxygen (O_2_) consumed, and carbon dioxide (CO_2_), metabolic heat (H_prod_) and sweat produced, during a *single* bout (30-min at 75% VO_2max_) of aerobic countermeasure exercise by theoretical female and male (in brackets) astronaut populations (see^9^).

Stature (m)	1.50	1.60	1.70	1.80	1.90
EE (MJ)	1.01 (1.28)	1.15 (1.45)	1.30 (1.64)	1.46 (1.84)	1.62 (2.05)
O_2_ (L)	49.3 (61.7)	56.1 (70.2)	63.4 (79.3)	71.0 (88.9)	79.2 (99.0)
CO_2_ (L)	42.8 (55.4)	48.7 (63.1)	55.0 (71.2)	61.7 (79.8)	68.7 (88.9)
H_prod_ (kJ)	952 (1200)	1084 (1366)	1223 (1542)	1371 1729)	1528 (1926)
Sweat (mL)	216 (303)	250 (350)	288 (401)	328 (455)	371 (513)

See main text for definition of assumptions.

### 24-h values

For all 24-h parameters at all statures, estimates for females were lower than comparable estimates for theoretical male astronauts^9^ (Table 3). In theoretical female astronauts, the increase in body size alone from 1.50-m to 1.90-m increased 24-h TEE by + 30% (8.0–10.4-MJ) *vs*. + 44% (8.9–12.9-MJ) in males, O_2_ consumption by + 60% (340–545-L) *vs*. + 60% (397–636-L) in males, CO_2_ production by + 60% (268–430-L) *vs*. + 60% (313–501-L) in males, heat production by + 60% (5.0–8.0-MJ) *vs*. + 60% (5.7–8.0-MJ) in males, and water requirements by + 17% (2.51–2.94-L) *vs*. + 19% (2.63–2.94-L) in males. With aerobic CM exercise, these increases (from a stature of 1.50-m to 1.90-m) were: *24-h TEE*: + 25% (8.0–10.0-MJ) to 31% (10.4–13.6-MJ) in females *vs*. + 29% (8.9–11.9-MJ) to 32% (12.9–17.0-MJ) in males; *O*_*2*_: + 29% (340–438-L) *vs*. + 31% (397–494-L) in males; *CO*_*2*_: +32% (268–353-L) *vs*. + 35% (501–679-L) in males; *H*_*prod*_: + 38% (5.7–8.1-MJ) *vs*. + 42% (9.1–13.0-MJ); *Water Requirements*: + 17% (2.51–2.94-L) to + 25% (2.94–3.69-L) in females *vs*. + 23% (2.63–3.23-L) to + 33% (3.13–4.16-L) for males^9^.

**Table 3:** A 24-h values for theoretical female astronaut populations without, and with, the use of ISS-like countermeasure (CM) exercise (modelled as *two* bouts of 30-min of cycle ergometry at 75% VO_2max_) and (in brackets) comparable values from the theoretical male astronaut populations (see ^9^).

Stature (m)	1.50	1.60	1.70	1.80	1.90
Without CM exercise					
TEE (MJ)	8.0 (8.9)	8.5 (9.9)	9.1 (10.8)	9.7 (11.9)	10.4 (12.9)
O_2_ (L)	340 (397)	387 (451)	436 (510)	489 (571)	545 (636)
CO_2_ (L)	268 (313)	305 (356)	344 (401)	386 (450)	430 (501)
H_prod_ (MJ)	5.0 (5.7)	5.7 (6.5)	6.4 (7.3)	7.2 (8.2)	8.0 (9.1)
Water requirements (L)	2.51 (2.63)	2.61 (2.74)	2.71 (2.86)	2.82 (2.99)	2.94 (3.13)
With CM exercise					
TEE (MJ)	10.0 (11.9)	10.8 (12.8)	11.7 (14.1)	12.6 (15.5)	13.6 (17.0)
O_2_ (L)	438 (494)	499 (562)	563 (634)	631 (711)	703 (792)
CO_2_ (L)	353 (423)	402 (482)	454 (544)	507 (610)	567 (679)
H_prod_ (MJ)	6.9 (8.1)	7.8 (9.2)	8.8 (10.4)	9.9 (11.6)	11.0 (13.0)
Water requirements (L)	2.94 (3.23)	3.11 (3.44)	3.29 (3.67)	3.49 (3.90)	3.69 (4.16)

*TEE* total energy expenditure, *O*_*2*_ total oxygen consumed, *CO*_*2*_ total carbon dioxide produced, *H*_*prod*_ total metabolic heat produced. See main text for definition of assumptions.

**Figure 1:**
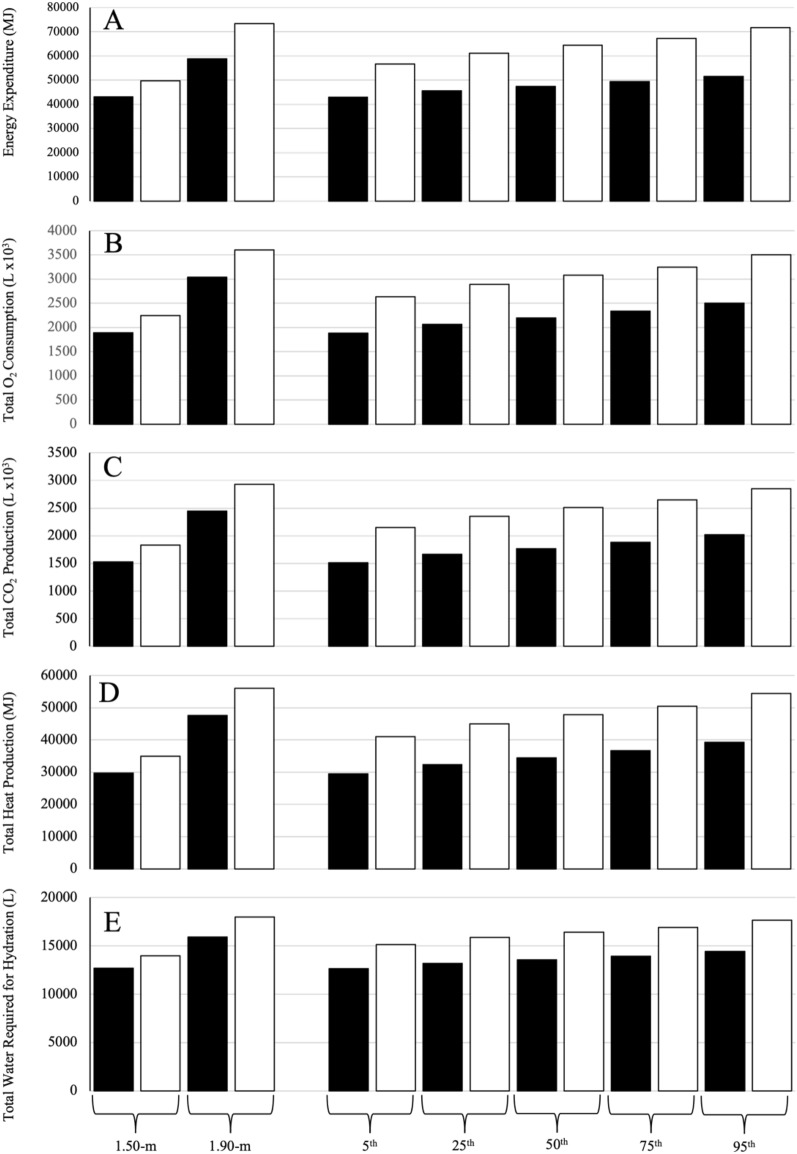
Energy expended (**A**), oxygen (**B**) consumed, carbon dioxide (**C**) and heat (**D**) produced, and water required for hydration (**E**) for all-female (black bars) and all-male (white bars, see^9^ crews of different statures during a 1080-day mission using aerobic countermeasure exercise. The left portion of the figure shows female and male data when compared at absolute statures of 1.50-m and 1.90-m, the right portion shows data when compared at the 5th, 25th, 50th, 75th and 95 percentile for stature for United States (US) females and males based on the US Centre for Disease Control (CDC) 2015–2016 National Health and Nutrition Examination Survey (NHANES)^28^.

Compared with a ‘small-sized’ (all individuals with a stature of 1.50-m) all-female crew performing no exercise, a ‘large-sized’ (all individuals with a stature of 1.90-m) crew performing ISS-like aerobic CM exercise require an additional 678-MJ of energy (288-MJ [for the effect of stature alone from 1.50-m to 1.90-m] plus 390-MJ [for the effect of CM exercise at a stature of 1.90-m]). The ‘large-sized’ crew performing CM exercise would also consume an additional 43.6 × 10^3^-L O_2_ (24.6 x 10^3^-L plus 19.0 x 10^3^-L), produce an additional 35.9 × 10^3^-L CO_2_ (19.4 x 10^3^-L plus 16.5 x 10^3^-L) and 727-MJ of metabolic heat (360-MJ plus 367-MJ), and consume an additional 141-L of water (51.9-L plus 89.0-L) for hydration per month (Table 4). The effects of increasing stature and the use of CM exercise in an all-female, four-person, crew during missions of 30-, 90-, 180-, 360-, 720- and 1080-days, are shown in the Supplementary Material (Figs. 1 Supp–5 Supp).

**Table 4:** Absolute increase in energy expended, oxygen (O_2_) consumed, carbon dioxide (CO_2_) and heat produced (H_prod_), and water required for hydration, during 30-day and 1080-day missions resulting from the increase in body size *alone* between a ‘small-sized’ (all individuals with a stature of 1.50-m) and ‘large-sized’ (all individuals with a stature of 1.90-m) female crew, and the use of aerobic countermeasure (CM) exercise.

	Increased (1.50- to 1.90-m) body size (without CM exercise)		Use of CM Exercise*	
	30-day	1080-day	30-day (1.50–1.90-m)	1080-day (1.50–1.90-m)
Energy (MJ)	+ 288 (+ 475)	+ 10,364 (+ 17,083)	+ 243–390 (+ 306–491)	+ 8749–14,037 (+ 11,019–17,680)
O_2_ (L × 10^3^)	+ 24.6 (+ 28.8)	+ 887 (+ 1036)	+ 11.8–19.0 (+ 14.8–23.8)	+ 426.3–683.9 (+ 533–856)
CO_2_ (L × 10^3^)	+ 19.4 (+ 22.7)	+ 699 (+ 816)	+ 10.3–16.5 (+ 13.3–21.3)	+ 370–593.6 (+ 479–768)
H_prod_ (MJ)	+ 360 (+ 412)	+ 12,968 (+ 14,832)	+ 229–367 (+ 288–462)	+ 8228–13,202 (+ 10,371–16,640)
Water (L)	+ 51.9 (+ 60.5)	+ 1867 (+ 2180)	+ 51.8–89.0 (+ 72.7–123.1)	+ 1864–3205 (+ 2619–4432)

In brackets, comparable values from the theoretical male astronaut populations (see^9^).

*A range of values is presented for ‘Use of CM Exercise’ data because the magnitude of the effect of CM exercise increases with increasing stature.

### Comparing theoretical female and male astronaut populations

At the lower (1.50-m) end of the comparison range, relative (%) differences between theoretical female and male astronauts^9^ ranged from − 5% for basal fluid needs up to − 29% for water loss through sweating during a single bout of aerobic CM exercise (Table 5). These differences were comparable at the upper (1.90-m) end of the comparison range, except for resting metabolic rate (RMR) (− 12% *vs*. − 21%), and 24-h EE without (− 10% *vs*. − 19%), and with (− 13% *vs*. − 20%), CM exercise, which were all markedly lower in females.

**Table 5:** Relative (%) difference between the theoretical female and male astronaut populations (see^9^) at the lower (1.50-m) and upper (1.90-m) ends of the stature comparison range, and at the 50th percentile for stature for United States females (1.615-m) and males (1.757-m).

Stature (m)	Relative (%) difference of females from males		
	1.50-m	1.90-m	50th Percentile*
Characteristics			
Body mass (kg)	− 14	− 14	− 28
VO_2max_ (L·min^−1^)	− 20	− 20	− 32
RMR (MJ·day^−1^)	− 12	− 21	− 27
Basal M_prod_ (J·s^−1^)	− 13	− 13	− 26
Basal fluid needs (L·d^−1^)	− 5	− 6	− 11
1 × bout of aerobic CM exercise			
EE (MJ)	− 21	− 21	− 33
O_2_ (L)	− 20	− 20	− 33
CO_2_ (L)	− 23	− 23	− 35
H_prod_ (MJ)	− 21	− 21	− 33
Water requirements (L)	− 29	− 28	− 41
24-h values without CM exercise			
EE (MJ)	− 10	− 19	− 25
O_2_ (L)	− 14	− 14	− 28
CO_2_ (L)	− 14	− 14	− 28
H_prod_ (MJ)	− 12	− 12	− 26
Water requirements (L)	− 5	− 6	− 11
24-h values with CM exercise			
EE (MJ)	− 13	− 20	− 26
O_2_ (L)	− 16	− 16	− 29
CO_2_ (L)	− 17	− 17	− 29
H_prod_ (MJ)	− 15	− 15	− 28
Water requirements (L)	− 9	− 11	− 18

*VO*_*2max*_ maximal rate of oxygen uptake, *RMR* resting metabolic rate, *M*_*prod*_ metabolic heat production, *EE* energy expenditure, *O*_*2*_ total oxygen consumed, *CO*_*2*_ total carbon dioxide produced, *H*_*prod*_ total metabolic heat produced. *For United States (US) females (1.615-m) and males (1.757-m) based on the US Centre for Disease Control (CDC) 2015–2016 National Health and Nutrition Examination Survey (NHANES)^28^.

When compared at the 50th percentile for stature for US males and females based on the US Centre for Disease Control (CDC) 2015–2016 National Health and Nutrition Examination Survey (NHANES)^28^, these differences were markedly larger than when compared at the lower (1.50-m) and upper (1.90-m) end of the stature comparison range for all measures, ranging from − 11% for basal fluid needs to − 41% for fluid requirements for one bout of CM exercise (Table 5). The magnitude of these differences was consistent from the 5th to the 95th percentile based on US CDC NHANES data. Consistent with the 24-h differences (Table 5), absolute EE, O_2_, CO_2_, H_prod_ and fluid requirements for a 4-person all-female crew were lower than for an all-male^9^ crew during a 1080-day mission using CM exercise (Fig. 1) with greater differences at the percentile comparisons compared with those at absolute statures.

## Discussion

Building upon an approach developed to estimate the effect of body ‘size’ and CM exercise upon TEE, O_2_ consumption, CO_2_ and metabolic heat production, and water requirements, in theoretical male astronauts^9^, with where appropriate and available, female specific equations, this paper provides the first estimates of these parameters for theoretical all-female crews. As in the previous study on theoretical male astronauts^9^, body ‘size’ was indexed from varying stature rather than body mass, as total stature is the only anthropometric criteria against which astronauts for ESA, NASA and CSA are currently selected. In addition, by using the same approach, this study enables a qualitative comparison with theoretical male astronauts from Scott et al.^9^ based on available ISS astronaut physical (stature, body mass and body mass index [BMI]) and physiological (relative VO_2max_) data. For all parameters at all statures, estimates for theoretical females were lower than for comparable male astronauts across a stature range of 1.50- to 1.90-m, and were more than 25% lower for most measures when compared at the 50th percentile for stature for US females (1.615-m) and US males (1.757-m)^28^.

As discussed in the paper of Scott et al.^9^, although closed-loop regenerative O_2_, water and CO_2_ management systems will reduce the absolute magnitude of some of the effects of body size and CM exercise, the effect on metabolic energy requirements has direct implications on the supply (and potential re-supply), storage and disposal (of packaging) of food. Based on the volume of a single 1.53-MJ portion of thermostabilized ‘ready-to-eat’ food (weight 250-g, energy: 365-kcal [1.53-MJ]; energy density: 1.46-kcal·g^−1^; volume: 340-cm^3^), the study of Scott et al.^9^ estimated that the combined effects of body size and CM exercise in a theoretical all-male crew would require the additional 5688-kg of food occupying 7.7-m^3^ during a 1080-day mission. In comparison, an all-female crew would require an additional 3993-kg of food occupying 5.4-m^3^ during a mission of identical length, savings of 1695-kg and 2.3-m^3^, the latter equivalent to approximately 4% of the habital volume (i.e., 60-m^3^) of a ‘Gateway’ module, which would be operationally highly significant.

To meet the calorie needs of astronauts in future Long-duration Exploration Missions (LDEMs), where re-supply will be extremely challenging, novel in-flight food production systems may serve to reduce the mass and volume of food that must be launched and stored with the crew. Technologies that could be the basis of such systems include the direct culture of animal cells (‘cultivated’ or ‘cultured’ meat)^29^, the use of hydrogen-oxidizing bacteria, and the cultivation of microalgae^30^. Protein is the major output of such systems and, given that the protein requirement for female and male astronauts is currently 0.8-g·kg^−1^ body mass^31^, a ‘smaller’ crew would, therefore, reduce system requirements (to meet daily protein need) and system resources (to meet the total mission protein requirement). Assuming 50th percentile US female and male stature (resulting in body masses of 59.2- and 81.8-kg), and that such a system is required to provide up to two-thirds of the daily protein requirement (the current defined maximum dietary contribution of animal protein)^31^, an all-female crew would require only 72% of the protein per day required for a four-person all-male crew (127- *vs*. 175-g·day^−1^), corresponding to 52.3-kg less (137.1-kg *vs*. 189.4-kg) protein over a 1080-day mission. Were the daily protein requirement to be increased to the upper end (1.8-g·kg^−1^ body mass) of the range proposed for future LDEMs^32^, the difference in protein requirements required for an all-female crew would correspond to 117.7-kg less (308.4-kg *vs*. 426.2-kg) protein over a 1080-d mission.

Due to the constraints of exploration vehicles, optimized (i.e., more effective and efficient) exercise CMs are required for future LDEMs^33^. Optimization also includes minimal utilization of mission and life support system resources, and particularly food, as routine and off-nominal mission tasks appear to be an important component of overall in-flight physical activity and TEE^6^. Numerous experimental CM exercise strategies have been evaluated in terms of ameliorated de-conditioning induced by long-term head down tilt bed rest—the most commonly used ground-based analogue of microgravity—with high-intensity jumping^34,35^ and high-intensity, low-volume cardiorespiratory interval training^36^ showing promise by virtue of appearing relatively effective across a range of outcomes, but requiring substantially less exercise time than that currently prescribed on ISS^3^, and thus calorific, and other potential life-support requirements. Jump-based training is yet to be evaluated in microgravity on the ISS^37^, but the results of the experimental ISS ‘SPRINT’ exercise program, based on high-intensity, low-volume interval training, recently reported comparable physiological outcomes to the current ISS CM exercise programme, but required 33% less time overall, and aerobic and resistance exercise volumes were reduced by 17% and 34–44% respectively^38^. Thus, although energy expenditure associated with the SPRINT protocol has yet to be quantified, presumably reduced exercise time and volume results in a lower CM exercise energy expenditure and overall metabolic activity.

Independent of energy and water requirements, and metabolic activity, stature itself may already be a factor in future human exploration mission design and planning. The ISS modules have a diameter of approximately 4.2-m^39^, with the internal ‘diameter’ (i.e., that available for work and translation through ISS) being smaller due to the internal racks (e.g., racks consume approximately 25-m^3^ of the 75-m^3^ internal volume of ESA's Columbus Module). This allows crewmembers to adopt a ‘vertical’ orientation (i.e., with the human body vertical axis perpendicular to the long axis of the modules) and work ‘shoulder-to-shoulder’ (or back-to-back) with each other whilst allowing translation and emergency egress. However, the diameter of modules for the forthcoming ‘Gateway’ that will orbit the Moon^40^ will be only 3-m^41^, meaning that taller (i.e., those in the upper stature percentiles) crew are unlikely to be able to ‘stand’ within the internal volume when orientated vertically. This may well result in a decision to adopt a ‘horizontal’ orientation^42^ (i.e., with the body’s vertical axis parallel to the module long axis), with crew members working and translating ‘head-to-foot’. Whilst this approach would facilitate crew of similar stature to current guidelines, it could result in poorer workspace ergonomics. As such, crews composed of individuals with smaller statures may also have potential habitability benefits (i.e., perceived volume and sensation of ‘crowdedness’) when co-working in the same module^43^. In addition, there may also be reduced crew "traffic interactions", where crewmembers need to move to allow another to pass and/or share equipment or workstations^43^.

As a result of the relatively (compared to males) low number of female astronauts, the physical and physiological characteristics used as the basis for the calculations in this paper were taken from a small (n = 7) group published by Moore et al.^18^. This group had a mean stature (1.693-m) and body mass (65.0-kg) equivalent to 19th the 30th percentiles respectively for US females^28^, giving a mean BMI of 22.7-kg·m^−2^, equivalent to the 18th percentile for US females^28^. In comparison, the male astronauts (n = 30) in the study of Moore et al.^18^ whose data were used by Scott et al.^9^ had a mean stature of 1.755-m and body mass of 81.6-kg, equivalent to the 51st and 40th percentiles for US males, resulting in a mean BMI of 26.5-kg·m^−2^ (36th percentile for US males)^28^. As such, it is possible that this small group is not truly representative of the ISS female astronaut population (38 individuals as of March 2021)^44^.

Standardised (1.50-m, 1.60-m, 1.70-m, 1.80-m, 1.90-m) stature intervals across the range accepted by the ESA, NASA and CSA for astronaut recruitment were used to make a direct comparison between theoretical female and male astronauts. However, a stature of 1.50-m and 1.90-m represent the extremes for the US populations: a stature of 1.50-m is 6.3-cm below the 1st percentile for males and 1.90-m is 12.9-cm above the 99th percentile for females^28^. Thus, females were qualitatively compared with males at fixed stature percentiles for US females and males based on data from the US CDC's 2015–2016 NHANES^28^. This approach augmented the tendency for lower resource requirements for females, with the greatest differences being predicted in the response to simulated aerobic CM exercise (Table 5). Based on a comparison at the 50th percentile for stature and utilising daily CM exercise, per day, the theoretical female astronaut population required 26% less energy, 29% less O_2_ and 18% less water for hydration, and produced 29% less CO_2_ and 28% metabolic heat.

The study has a number of limitations common with those described in the study of Scott et al.^9^ based on theoretical male astronauts. Specifically: (1) the need to make a number of assumptions about the components of TEE in microgravity in the absence of space-specific equations and data; (2) the possibility that the elevated (~ 0.5%) atmospheric CO_2_ might have a stimulatory effect on metabolism^45^; (3) the necessity to model resistance exercise as a second bout of aerobic exercise (in the absence of appropriate validated equations for calculation of energy expenditure and water requirements), and; (4) the equation used to predict exercise sweat losses (based on estimates of the clothing biophysical properties) was not explicitly defined for use in microgravity. Furthermore, an additional limitation, also described in Scott et al.^9^, is that given the unavailability of individual astronaut data, geometric similarity in terms of VO_2max_ between the different body sizes was assumed, which may not be the case^46,47^ and could have resulted in an underestimation of metabolism and energy expenditure in the larger theoretical female populations. However, the purpose of this paper was to make a direct comparison with the results from theoretical male astronauts^9^ and this issue will be addressed in subsequent papers for both male and female theoretical populations using multi-parameter allometric scaling techniques.

## Conclusion

Using published anthropometric (stature, body mass and BMI) and physiological (relative VO_2max_) characteristics of female ISS astronauts, this theoretical study based on stature as the key operational criterion has, for the first time, provided estimates of energy expenditure, O_2_ use, CO_2_ and metabolic heat production, and water requirements for hydration, for an all-female crew spanning a stature range of 1.50–1.90-m during exploration missions without, and with, the use of ISS-like aerobic CM exercise. Compared with a ‘small-sized’ (1.50-m) crew without CM exercise, a ‘large-sized’ (1.90-m) all-female crew exercising would require an additional 678-MJ of energy, 43.6 × 10^3^-L of O_2_ and 141-L of water, and produce an additional 35.9 × 10^3^-L of CO_2_ and 727-MJ of heat each month. All parameter estimates were qualitatively lower than those for a theoretical all-male crew from a previous study based on a similar methodology, with relative differences at equivalent statures ranging from − 5% to − 29%, and increasing to − 11% to − 41% when compared at the 50th percentile for stature for US females and males. The increase in TEE with increasing body size and use of aerobic CM exercise was markedly less in females compared with males, resulting in less additional food (+ 3993- *vs*. + 5688-kg) and storage volume (+ 5.4- *vs*. + 7.7-m^3^) required to meet energy requirements during a 1080-day mission. These estimated differences result from lower resting and exercising O_2_ requirements (based on available astronaut data) of theoretical female astronauts, who are lighter than theoretical male astronauts at equivalent statures and have lower relative VO_2max_ values. These data, combined with the move towards smaller diameter space habitat modules, suggest that there may be a number of operational advantages to all-female crews during future human space exploration missions.

## Methods

This study was theoretical and did not involve any experimentation with human volunteers. The calculations performed were based on published data from female astronauts and established physiological equations. As such, ethical approval for the study was not required.

### Assumptions and rationales

For calculation purposes, and to provide upper and lower limits for the estimation of the effect of body size, the following assumptions have been made in line with that employed previously to evaluate male-only crew^9^ with modifications, where appropriate, for female crew.

#### Assumptions about the missions and vehicle/habitat

1. LDEM durations will range from 30-day (transit-out, Lunar orbit, transit-back) up to 1080-day (transit-out, prolonged Martian orbit, transit-back), all without human surface exploration (i.e., crew will remain inside vehicles/habitats for the entire mission).
2. LDEMs will be crewed by four female astronauts.
3. The environment inside the vehicle is comparable to that currently on ISS (760-mmHg barometric pressure, 20.9% O_2_, ~ 0.5% CO_2_, approximately 79% nitrogen [N_2_] at 101.3-kPa [14.7-psi], mean temperature 22 °C, 55% relative humidity)^48,49^.
4. The slightly elevated CO_2_ concentration inside the space vehicle (compared to sea-level) as maintained on the ISS has no effect on metabolism, either at rest, or during exercise.
5. Airflow experienced by the crew member (provided by the vehicle/habitat ventilation system) during CM exercise is 0.5 m·s^−1^.

#### Assumptions about the crew and their physiology at rest

1. Crew stature ranges from 1.50 (‘small’) to 1.90-m (‘large’) (59.1–74.8″), which is representative of historical and current stature requirements (for both males and females) for NASA^50^, and the European (ESA)^51^ and Canadian (CSA) Space Agencies^52^. For simplicity all individuals within the same crew are assumed to be of identical stature.
2. Crew are geometrically scaled (i.e., that all dimensions change proportionally among all individuals) across the defined stature range.
3. Independent of stature, all crew have, and maintain, a BMI of 22.7-kg m^−2^, calculated from the mean stature (1.693-m) and body mass (65.0-kg) of a group of seven female ISS astronauts^18^.
4. Crew are all 40 years old (for the purposes of calculation), accounting for the prolonged training likely required for an LDEM^9^. Crew would, of course, age during LDEMs and this would influence the estimation of RMR (see *Calculations* below). However, this effect is minor. Increasing only age from 40- to 43-years old, decreases RMR by only 13-kcal, or, with the assumptions and calculations used in this paper, − 1.1% of estimated RMR at a stature of 1.50-m, decreasing to − 0.8% at a stature of 1.90-m.
5. As no 24-h TEE, or its components (RMR, and non-resting energy expenditure, composed of non-exercise activity thermogenesis [NEAT], exercise activity thermogenesis [EAT] and the thermic effect of food [TEF]) are currently available during spaceflight, the following are assumed:
  - RMR is equivalent to that on Earth, and does not change during the mission.
  - NEAT is minimal but not negligible, resulting in a PAL of 1.4 (equivalent to a very sedentary lifestyle on Earth) excluding CM exercise (see below for assumptions/calculations related to EAT for astronauts performing CM exercise).
  - EAT is related to the use of CM exercise (see below).
  - TEF requires 206-kcal (0.87-MJ) of energy expenditure^53^.
6. VO_2_ at rest (RMR) is 3.3-mL·kg^-1^·min^−1^^54^.
7. Respiratory exchange ratio (RER) at rest is 0.788^55,56^
8. Based on a resting VO_2_ of 3.3-mL·kg^−1^·min^−1^ and a resting RER of 0.788, resting VCO_2_ is 2.6-mL·kg^−1^·min^−1^.
9. Respiratory water losses are balanced by metabolic water production, and thus can be discounted, whereas transcutaneous water loss is only considered for total body water balance^57^.
10. Protein (95-g·day^−1^), sodium (4320-mg·day^−1^) and potassium (3062-mg·day^−1^) intake data are as reported from ISS Expeditions 26–37^58^.
11. Core temperature is 37 °C.

#### Assumptions about the crew and their physiology during exercise

Countermeasure exercise on ISS currently consists of two sessions per day (1 × 30–45-min of aerobic and 1 × 45-min of resistance), 6-day·week^−1^, with target workloads for steady-state and interval-type aerobic protocols of 75–80% and 60–90% VO_2max_ respectively^3^. However, due to the challenge of modelling non-steady state exercise^59,60^ such as intermittent, high intensity resistance exercise as used on ISS, for the purpose of this paper, CM exercise is modelled as 30-min of steady-state aerobic exercise at 75% VO_2max_, performed twice per day, 6-day·week^−1^. The assumptions used are as follows:

1. Crew have a relative VO_2max_ of 40.5-mL·kg^−1^·min^−1^, calculated from the mean body mass (65.0-kg) and absolute VO_2max_ (2.63-L·min^−1^) of a group of seven female ISS astronauts^18^.
2. During aerobic exercise crew wear light sports clothing (shorts, t-shirt and socks), with thermal and evaporative resistances equal to 0.06-m^2^ × °C/W and 0.01-m^2^ × kPa/W, respectively^61^.
3. VO_2_ during exercise is 30.4-mL·kg^−1^·min^−1^ (i.e., 75% of 40.5-mL·kg^−1^·min^−1^)^18^. For simplification, any warm-up or cool-down periods have been excluded.
4. RER during exercise at 75% VO_2max_ is 0.868, as meta-analysis reports that RER is consistently lower (− 0.03 RER units) in females^25^ compared with males, where in our previous study we assumed an RER of 0.898^62^. Menstrual cycle phase may influence whole-body substrate utilization during exercise, however, the majority of the literature reports no significant effect^63–65^, and given menstruation is typically suppressed in orbit^66^, no effect of the menstrual cycle is assumed.
5. Based on a VO_2_ of 30.4-mL·kg^−1^·min^−1^ and an RER of 0.868, VCO_2_ during exercise is 27.3-mL·kg^−1^·min^−1^.
6. Excess post-exercise oxygen consumption (EPOC), energy expenditure and VCO_2_ are 6% of that consumed during exercise^67^.
7. RER is equivalent to that at rest during recovery from exercise.

### Calculations

1. RMR was calculated using the Revised Harris–Benedict Equation^68^ for females, where:
  $$RMR \, \left( {{\text{kcal}}} \right) = \, 447.593 \, + \, \left( {9.247 \, \times {\text{ weight}}\;{\text{ in}}\;{\text{ kg}}} \right) \, + \, \left( {3.098 \, \times {\text{ height}}\;{\text{ in}}\;{\text{ cm}}} \right) \, - \, \left( {4.330 \, \times {\text{ age}}\;{\text{ in }}\;{\text{years}}} \right).$$
2. BMI^69^ was calculated as:
  $$BMI \, \left( {{\text{kg}} \cdot {\text{m}}^{-2} } \right) \, = {\text{ body }}\;{\text{mass}}\; \, \left( {{\text{kg}}} \right) \cdot {\text{height }}\;\left( {\text{m}} \right)^{ - 2}$$
3. Energy expenditure (EE) during exercise (kcal·min^−1^) was estimated using the Weir equation^70^, where:
  $$EE \, \left( {{\text{kcal}}} \right) \, = \, 3.94 \, \;{\text{VO}}_{2} \ ({\text{mL}}\cdot{\text{min}}^{ - 1} ) + \, 1.11{\text{ VCO}}_{2} \left( {{\text{mL}}\cdot{\text{min}}^{ - 1} } \right)$$
4. Body surface area (BSA) was calculated using the formula of Du bois and Du bois^71^:
  $$BSA \, \left( {{\text{m}}^{2} } \right) \, = \, 0.007184 \, \cdot {\text{height }}\;\left( {{\text{in}}\;{\text{ metres}}} \right)^{0.725} \cdot {\text{weight }}\left( {{\text{in}}\;{\text{kg}}} \right)^{0.425}$$
5. Insensible water needs (IWN), dietary solute load (DSL) and urine volume to maintain a 24-h urine volume at a concentration of 600-mmol·kg^−1^ (UV_600_)^72^ was calculated as:
  $$\begin{aligned} & IWN \, \left( {\text{L}} \right) \, = \, \left( {0.4 \, \cdot Energy \, \;Needs\;\left[ {{\text{in }}\;{\text{kcal}}} \right]} \right) \, / \, 1000 \\ & DSL \, \left( {{\text{mmol}}} \right) \, = \, \left( {Protein\; \, Intake \, \left[ {{\text{g}}\cdot{\text{d}}^{ - 1} } \right] \, / \, 0.175} \right) \, + \, \left( {2* \, \left( {Na \, \;Intake \, \left[ {{\text{mg}}\cdot{\text{d}}^{ - 1} } \right] \, / \, 23 \, + \, K \, \;Intake \, \left[ {{\text{mg}}\cdot{\text{d}}^{ - 1} } \right] \, / \, 39} \right)} \right) \\ & UV_{600} \left( {\text{L}} \right) \, = \, DSL \, / \, 600 \\ \end{aligned}$$
6. The rate of metabolic heat production (M_prod_) at rest was calculated as:
  $$M_{prod} \left( {{\text{J}} \cdot {\text{s}}^{ - 1} } \right) \, = \, \left( {VO_{2} \left[ {{\text{mL}}\cdot{\text{min}}^{ - 1} } \right] \, \times \, Thermal \, \;Equivalent \, \;of \, \;O_{2} \;at\; \, RER} \right) \, / \, 0.01433$$
  where the thermal equivalent of O_2_ at resting RER (0.788) is 4.788-kcal·min^−1^·L^−1^.
7. M_prod_ during exercise was calculated using the above formula, where the thermal equivalent of O_2_ at exercising RER (0.868) is 4.887-kcal·min^−1^·L^−1^.
8. Using an M_prod_ adjusted for the rate of external work during cycling (M_prod_ – Rate of External Work [W]), where W was assumed to be 20% of M_prod_ for non-cyclists^73^, fluid requirements during exercise were calculated by first, using partitional calorimetry formulae for body heat balance to derive the requirement for evaporative cooling (E_req_) and the maximal evaporative capacity of the environment (E_max_)^74^. The equation of Gonzalez et al.^75^ was then applied to estimate steady-state exercise sweating rate. No allowance was made for the thermal inertial lag in sweating onset as it is generally balanced by the reciprocal thermal decay post-exercise^76^.

Finally, as the standardised (1.50-, 1.60-, 1.70-, 1.80- and 1.90-m) stature intervals used in the paper of Scott et al.^9^ on theoretical male astronauts represent the extremes of the male (lower) and female (upper) stature range, for a more ecologically valid comparison, theoretical female and male^9^ populations were compared qualitatively using the 5th, 25th, 50th, 75th and 95th stature percentiles (female statures: 1.495-, 1.566-, 1.615-, 1.667- and 1.725-m; male statures: 1.625-, 1.702-, 1.757-, 1.804-, and 1.873-m) from adults in the US CDC's 2015–2016 NHANES^28^.

All calculations were performed using Microsoft Excel Version 16.62.

## Supplementary Information

Supplementary Figures.

## Data Availability

All data used for the calculations used in this paper can be made available on request. Requests for the data should be addressed to J.P.R.S.
