# Supplementary material for: Effects of body size and countermeasure exercise on estimates of life support resources during all-female crewed exploration missions
Source: Sci Rep. 2023 Apr 12;13:5950. doi: 10.1038/s41598-023-31713-6 (PMC10097614; doi:10.1038/s41598-023-31713-6)
Supplement: Supplementary file 1 — Supplementary Figures. [file 41598_2023_31713_MOESM1_ESM.docx]

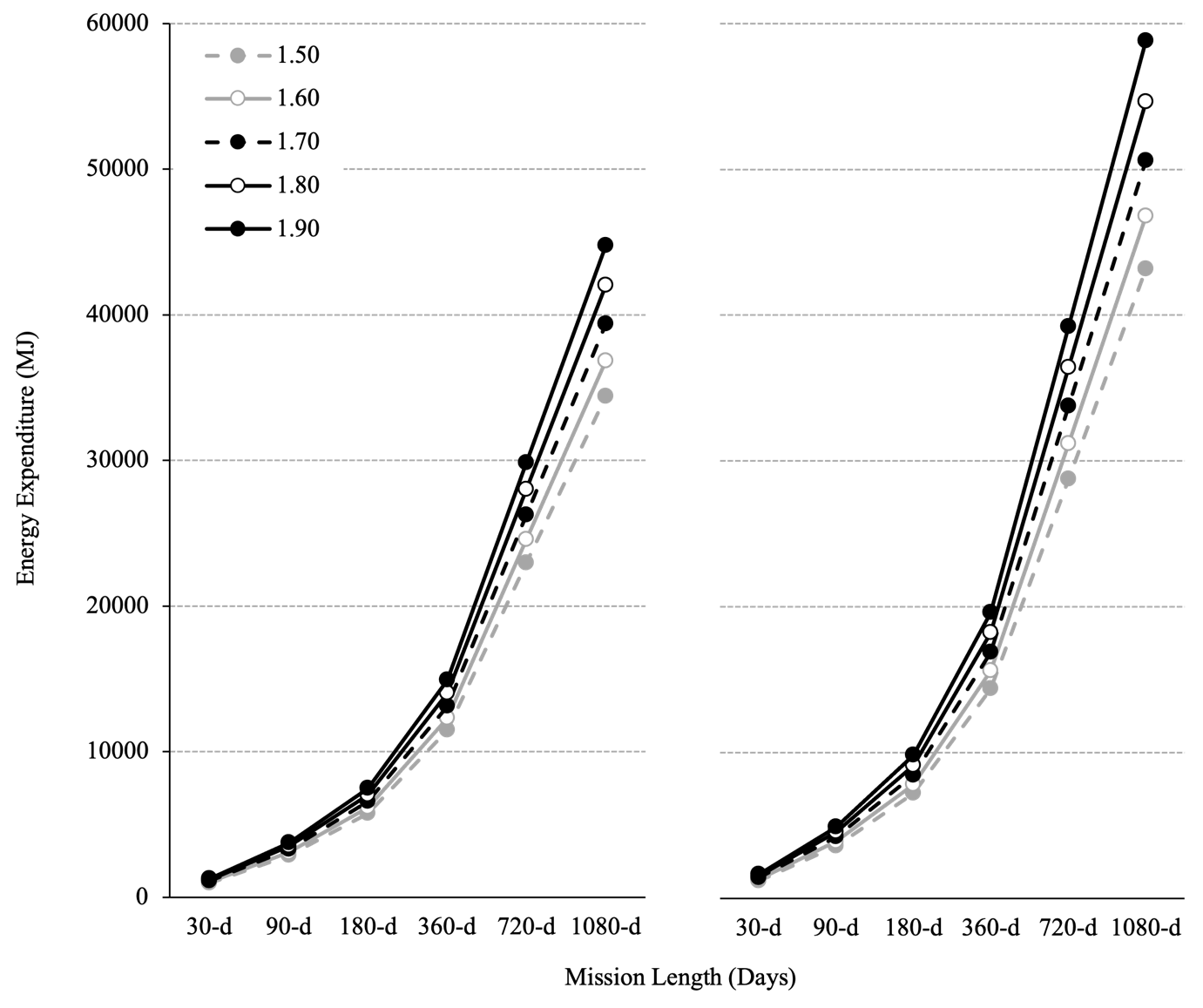


**Supplementary Figure 1:** Total energy expenditure (MJ) without (Left Panel) and with (Right Panel) countermeasure exercise during exploration missions of 30-, 90-, 180-, 360-, 720- and 1080‑d for a four-person crew based on theoretical female astronaut populations with statures of 1.50-m (broken grey line, filled circles), 1.60-m (solid grey line, open circles), 1.70-m (broken black line, filled circles), 1.80-m (solid black line, open circles) and 1.90-m (solid black line, filled circles).


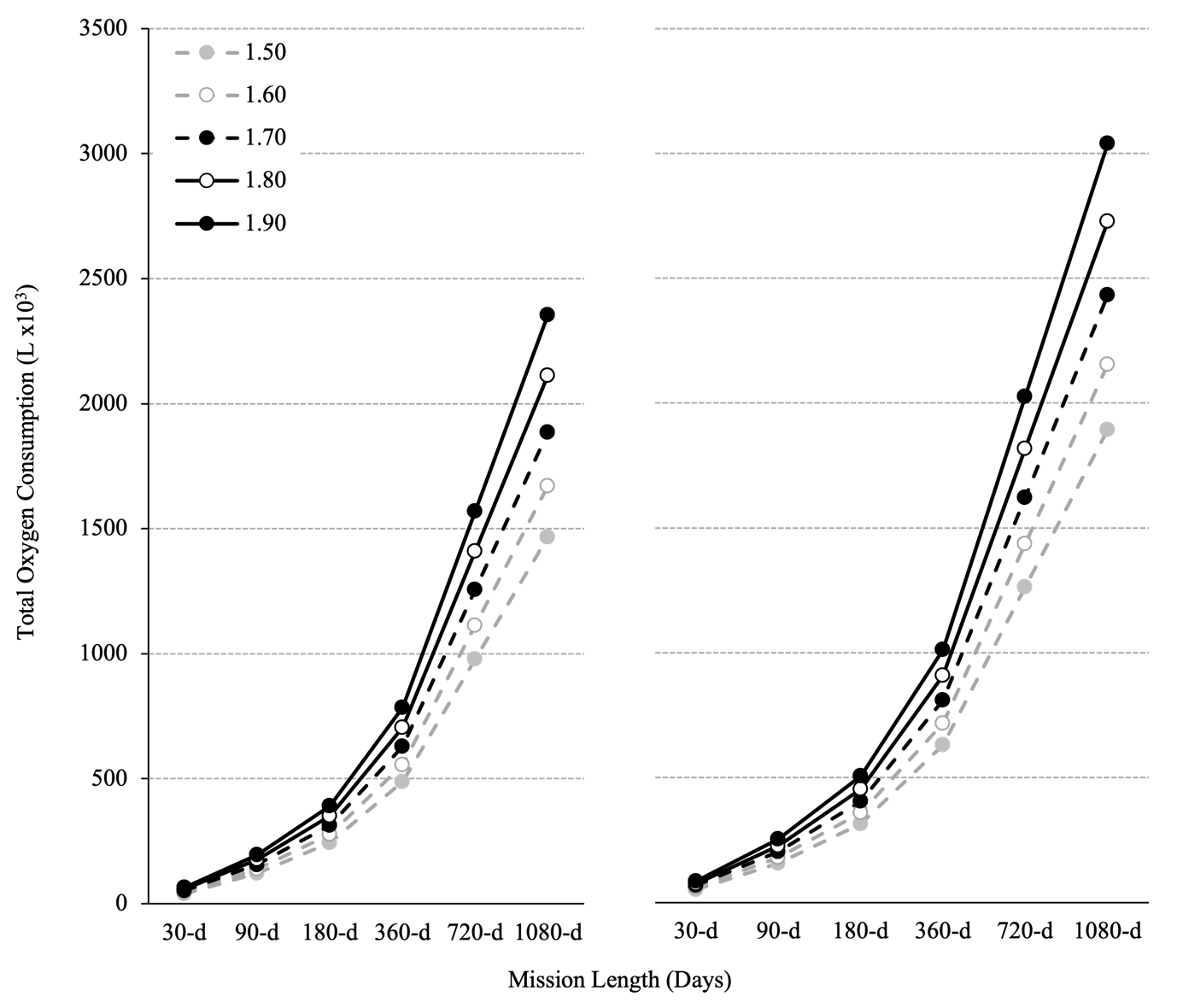


**Supplementary Figure 2:** Total oxygen consumption (x10^3^-L) without (Left Panel) and with (Right Panel) countermeasure exercise during exploration missions of 30-, 90-, 180-, 360-, 720- and 1080‑d for a four-person crew based on theoretical female astronaut populations with statures of 1.50-m (broken grey line, filled circles), 1.60-m (solid grey line, open circles), 1.70-m (broken black line, filled circles), 1.80-m (solid black line, open circles) and 1.90-m (solid black line, filled circles).

**
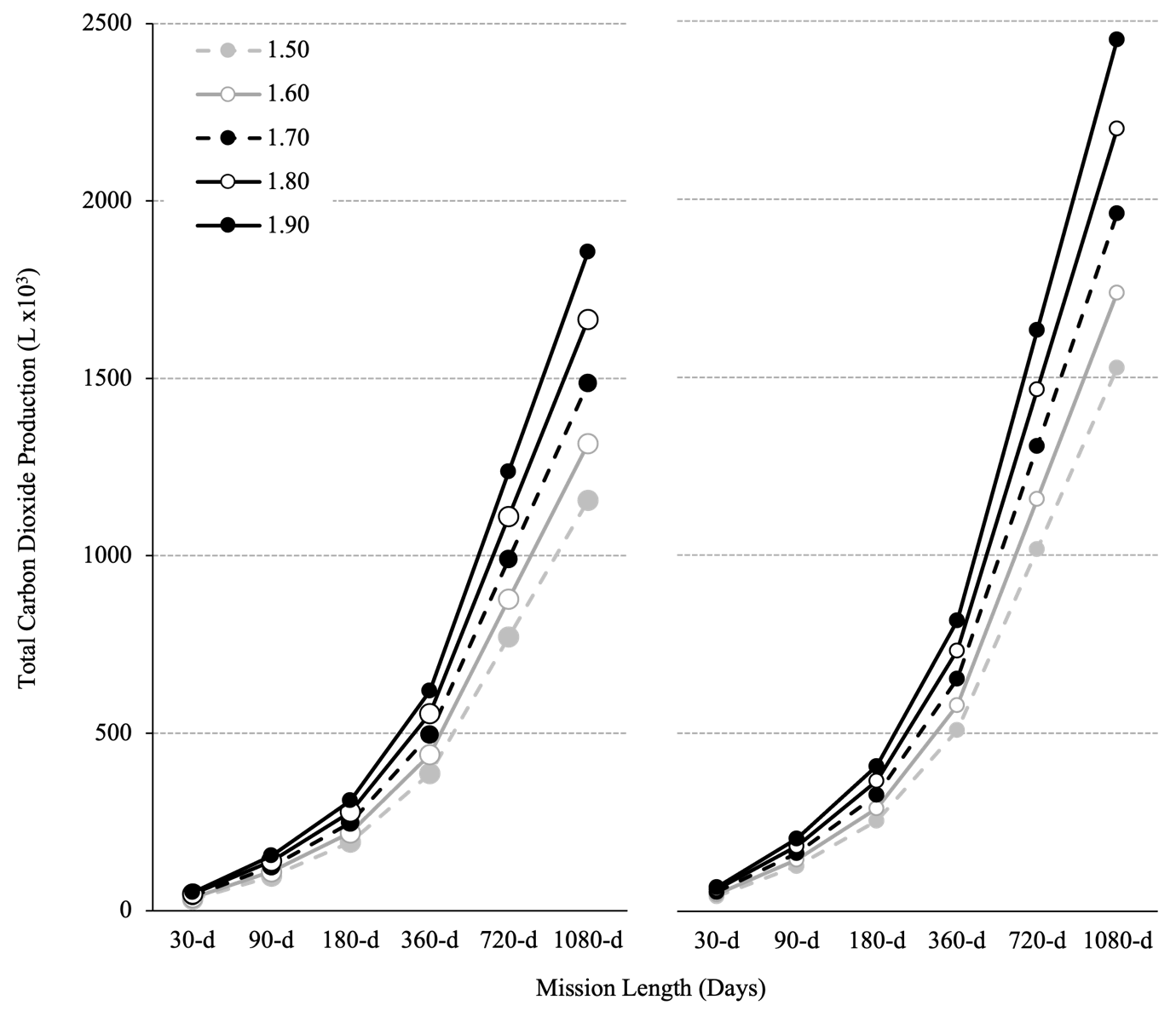
**

**Supplementary Figure 3:** Total carbon dioxide production (x10^3^-L) without (Left Panel) and with (Right Panel) countermeasure exercise during exploration missions of 30-, 90-, 180-, 360-, 720- and 1080‑d for a four-person crew based on theoretical female astronaut populations with statures of 1.50-m (broken grey line, filled circles), 1.60-m (solid grey line, open circles), 1.70-m (broken black line, filled circles), 1.80-m (solid black line, open circles) and 1.90-m (solid black line, filled circles).


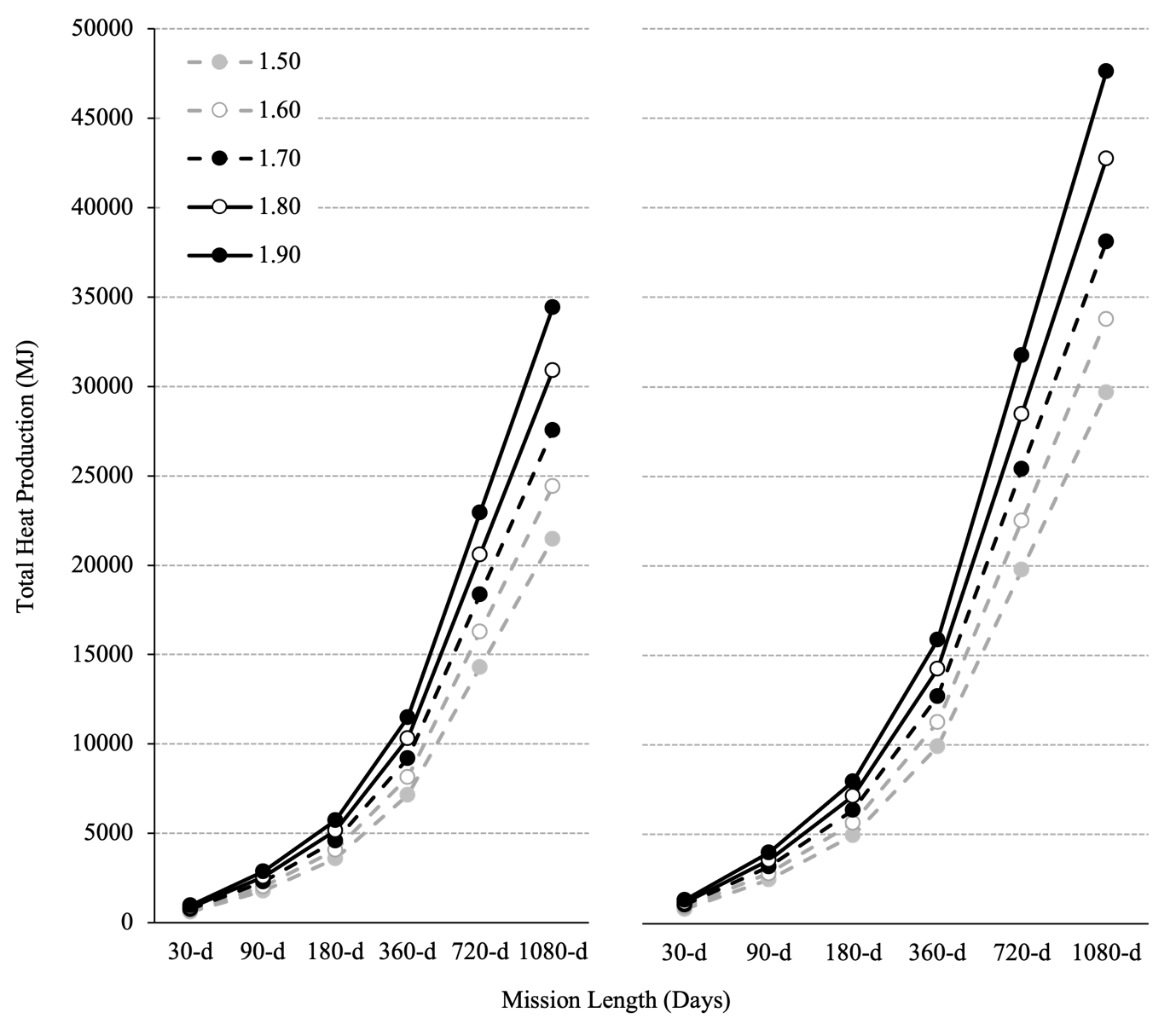


**Supplementary Figure 4:** Total metabolic heat production (MJ) without (Left Panel) and with (Right Panel) countermeasure exercise during exploration missions of 30-, 90-, 180-, 360-, 720- and 1080‑d for a four-person crew based on theoretical female astronaut populations with statures of 1.50-m (broken grey line, filled circles), 1.60-m (solid grey line, open circles), 1.70-m (broken black line, filled circles), 1.80-m (solid black line, open circles) and 1.90-m (solid black line, filled circles).


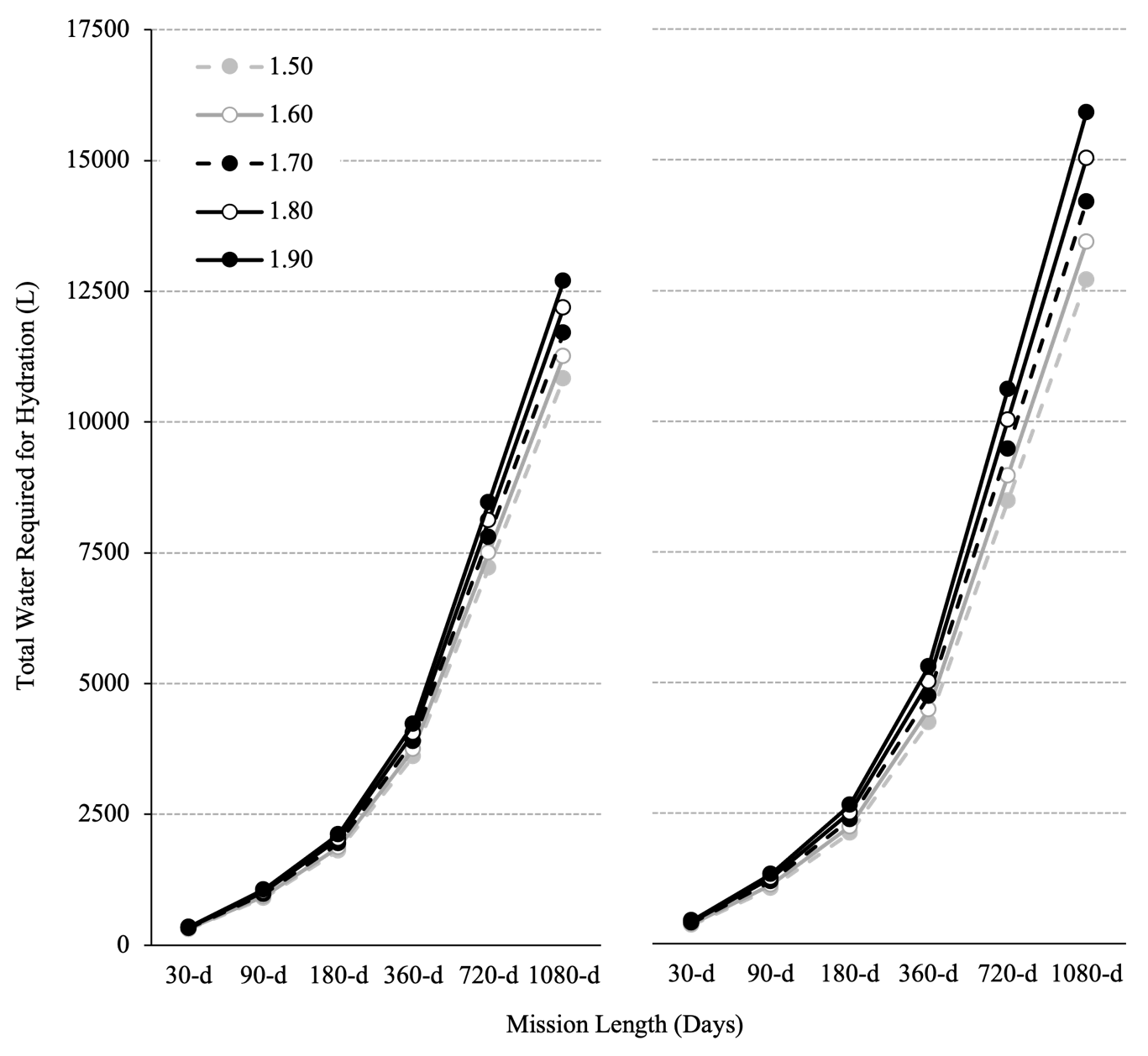


**Supplementary Figure 5:** Total water requirements (L) without (Left Panel) and with (Right Panel) countermeasure exercise during exploration missions of 30-, 90-, 180-, 360-, 720- and 1080‑d for a four-person crew based on theoretical female astronaut populations with statures of 1.50-m (broken grey line, filled circles), 1.60-m (solid grey line, open circles), 1.70-m (broken black line, filled circles), 1.80-m (solid black line, open circles) and 1.90-m (solid black line, filled circles).
